# Exploring Climate and Air Pollution Mitigating Benefits of Urban Parks in Sao Paulo Through a Pollution Sensor Network

**DOI:** 10.3390/ijerph22020306

**Published:** 2025-02-18

**Authors:** Patrick Connerton, Thiago Nogueira, Prashant Kumar, Maria de Fatima Andrade, Helena Ribeiro

**Affiliations:** 1 Programa de Pós-Graduação em Saúde Global e Sustentabilidade, Faculdade de Saúde Pública, Universidade de São Paulo, São Paulo 01246-904, Brazil; 2 Departamento de Saúde Ambiental, Faculdade de Saúde Pública, Universidade de São Paulo, São Paulo 01246-904, Brazil; thiagonogueira@usp.br (T.N.); lena@usp.br (H.R.); 3 Global Centre for Clean Air Research (GCARE), School of Engineering, Civil and Environmental Engineering, Faculty of Engineering and Physical Sciences, University of Surrey, Guildford, Surrey GU2 7XH, UK; p.kumar@surrey.ac.uk; 4 Institute for Sustainability, University of Surrey, Guildford, Surrey GU2 7XH, UK; 5 Departamento de Ciências Atmosféricas, Instituto de Astronomia, Geofísica e Ciências Atmosféricas, Universidade de São Paulo, São Paulo 05508-090, Brazil

**Keywords:** low-cost sensors, particulate matter, climate stressors, air quality, green–blue–gray infrastructure, cities

## Abstract

Ambient air pollution is the most important environmental factor impacting human health. Urban landscapes present unique air quality challenges, which are compounded by climate change adaptation challenges, as air pollutants can also be affected by the urban heat island effect, amplifying the deleterious effects on health. Nature-based solutions have shown potential for alleviating environmental stressors, including air pollution and heat wave abatement. However, such solutions must be designed in order to maximize mitigation and not inadvertently increase pollutant exposure. This study aims to demonstrate potential applications of nature-based solutions in urban environments for climate stressors and air pollution mitigation by analyzing two distinct scenarios with and without green infrastructure. Utilizing low-cost sensors, we examine the relationship between green infrastructure and a series of environmental parameters. While previous studies have investigated green infrastructure and air quality mitigation, our study employs low-cost sensors in tropical urban environments. Through this novel approach, we are able to obtain highly localized data that demonstrates this mitigating relationship. In this study, as a part of the NERC-FAPESP-funded GreenCities project, four low-cost sensors were validated through laboratory testing and then deployed in two locations in São Paulo, Brazil: one large, heavily forested park (CIENTEC) and one small park surrounded by densely built areas (FSP). At each site, one sensor was located in a vegetated area (Park sensor) and one near the roadside (Road sensor). The locations selected allow for a comparison of built versus green and blue areas. Lidar data were used to characterize the profile of each site based on surrounding vegetation and building area. Distance and class of the closest roadways were also measured for each sensor location. These profiles are analyzed against the data obtained through the low-cost sensors, considering both meteorological (temperature, humidity and pressure) and particulate matter (PM_1_, PM_2.5_ and PM_10_) parameters. Particulate matter concentrations were lower for the sensors located within the forest site. At both sites, the road sensors showed higher concentrations during the daytime period. These results further reinforce the capabilities of green–blue–gray infrastructure (GBGI) tools to reduce exposure to air pollution and climate stressors, while also showing the importance of their design to ensure maximum benefits. The findings can inform decision-makers in designing more resilient cities, especially in low-and middle-income settings.

## 1. Introduction

Ambient air pollution represents a critical public health issue throughout the world, having surpassed other factors in recent decades to become the main environmental exposure affecting human health [1]. Both air pollution and heat wave incidence have been associated with increases in all-cause mortality [2,3]. Even at low concentrations, exposure to air pollutants such as particulate matter (PM) has been linked to an increased risk of respiratory and cardiovascular diseases [4]. Increased epidemiological evidence for these relationships has resulted in a gradual reduction in recommended air pollutant levels from groups such as the World Health Organization [5]. Exposure to air pollution is especially critical in urban landscapes in low-and middle-income countries, where both population and emission sources tend to be more concentrated [6].

Although air pollution has become the primary environmental determinant of premature death, it should be noted that much progress has been made to curb air quality degradation. Especially in urban areas, efforts to limit concentrations of primary air pollutants have, in many cases, made significant strides [7,8]. Conversely, communities across the globe face increasing and uncertain threats from climate change, with vulnerable populations—especially those in the global south—facing disproportionate effects. Increased incidence and intensity of heat waves are particularly concerning for cities that already experience the urban heat island effect [9]. Research on emerging pollutants and better estimations of pollutant concentrations at high resolutions has also improved our understanding of the complexity of air pollution over this period. This development has coincided with the emergence of new, low-cost solutions for monitoring air pollutants, enabling levels of spatial and temporal resolution that were not previously possible [10]. These technologies also help us to better understand exposure to air pollution in near real-time and are especially important in poor and middle-income countries where monitoring stations are scarce.

Despite the challenges, city planners and policy-makers have an opportunity to reshape cities in the wake of a global pandemic [11]. Green–blue–gray infrastructure (GBGI) refers to natural features such as parks, wetlands or engineered greening that produce environmental benefits such as cooling, water management and leisure. Nature-based solutions such as GBGIs have emerged as valuable tools for mitigating exposure to air pollution [12], in addition to a multitude of other environmental benefits to communities [13,14]. Green structures such as tree rows act as a physical barrier to PM and also retain particles through deposition on leaf surfaces. In the case of temperature regulation, increasing vegetation coverage reduces temperatures while also working as natural sponges that provide protection against flooding. However, not all green infrastructure solutions offer similar benefits, and in some cases, factors such as decreased pollutant dispersion [15,16] and the potential of vegetation emission to form ozone [17] and fine particles [18] may offset or even surpass gains. Due to this range of possible outcomes, it is important to analyze exposure settings to understand the benefits GBGI interventions can offer. In this context, the present study explores the influence of green infrastructure in a tropical urban setting by examining air pollution and climate data collected using low-cost sensors at two locations in São Paulo, Brazil. The goal of this research is to demonstrate whether these features present any air quality or climate-mitigating benefits and the extent to which this can be measured.

This work aims to understand the intersection of air pollution and climate stressor mitigation via vegetation in an urban landscape. We employ low-cost sensors to compare roadside conditions to those within a park at two locations to estimate the potential mitigating impact of the vegetation. Low-cost sensors have not been deployed previously in São Paulo, making the data used here novel. The green infrastructure investigated here existed prior to the study—there was no intervention to introduce the GBGIs, offering a look at the real-world functionality of these tools.

## 2. Materials and Methods

After performance validation (see Section 2.2), the low-cost sensors were installed at two locations in Sao Paulo. The locations were selected due to their unique characteristics, containing a combination of green and built areas. Two sensors were installed at both sites, with one sensor being located in the middle of the vegetated area (hereafter “park” sensors) and the other being located adjacent to one of the bordering roads (hereafter “roadside” sensors). The data from these sensors were analyzed to assess the mitigating impact of the vegetation at each site. The RStudio package openair was used for quantitative analysis of the sensor data and generating graphs (https://cran.r-project.org/web/packages/openair/index.html (accessed on 23 September 2024)); QGIS was used for spatial analysis and mapping (https://qgis.org/ (accessed on 23 September 2024)); CloudCompare was used to analyze lidar data (https://www.cloudcompare.org/ (accessed on 23 September 2024)). From the cleaned and compiled data, the variation in the hourly average of all variables was calculated.

### 2.1. Site Description

São Paulo is a mega-city located in southeastern Brazil, with a population of 11.4 million (21 million when considering the metropolitan region composed of 39 municipalities). The city has a large and constantly evolving vehicle fleet, which represents a primary emission source affecting air quality in the city [19]. São Paulo is also subject to the contribution of regional area sources such as biomass burning, which has long been identified as a main emission source in Brazil [20]. While the city is composed primarily of densely built areas, vegetation is more dominant in some regions of the city compared to others [21]. In this context, urban parks can offer protection from climate stressors such as heat waves [22]. However, the characteristics of the park, such as the quantity and structure of vegetation, as well as how vegetation is orientated, determine the range of both thermal protection and PM reduction benefits it provides [23,24]. São Paulo has a humid subtropical climate, with little seasonal variation in the vegetation’s composition; thus, there is no need for a distinct analysis across seasons.

**Figure 1:**
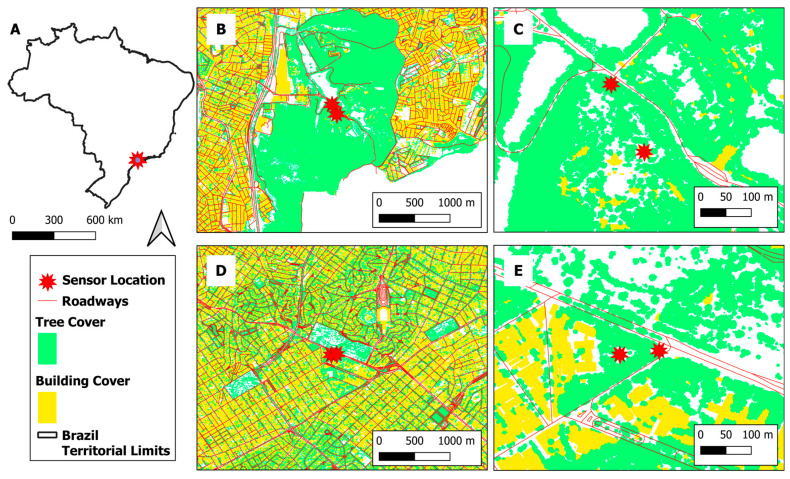
Study site location within Brazil (**A**) and presence of roadways, vegetation and built area at the CIENTEC (**B**,**C**) and FSP (**D**,**E**) sites.

The selection of sites where the sensors were installed, therefore, focused on two distinct urban park formats: a small green island vs. an extensive green area. Figure 1 shows the location and some characteristics of the two sites using lidar data that have been classified as vegetation and built area. The smaller park is part of the campus of the University of Sao Paulo’s School of Public Health (Faculdade de Saúde Pública—hereafter FSP). The FSP site covers an area of approximately 3 hectares and is positioned between two major arterial roads on the north and southeastern sides. The western area of the park contains the main university buildings, representing the tallest structures in the surrounding area. While cars are permitted within the park area, vehicle traffic is extremely limited, as onsite parking is reserved for authorized staff only. The larger park is the University of Sao Paulo’s Science and Technology Park (hereafter CIENTEC). The CIENTEC park is located within the Fontes do Ipiranga State Park, which covers an area of approximately 470 hectares. An arterial road runs through the park, connecting neighborhoods to the central region of the city. At both sites, a mix of vegetation lines the nearest roads at each site.

**Figure 2:**
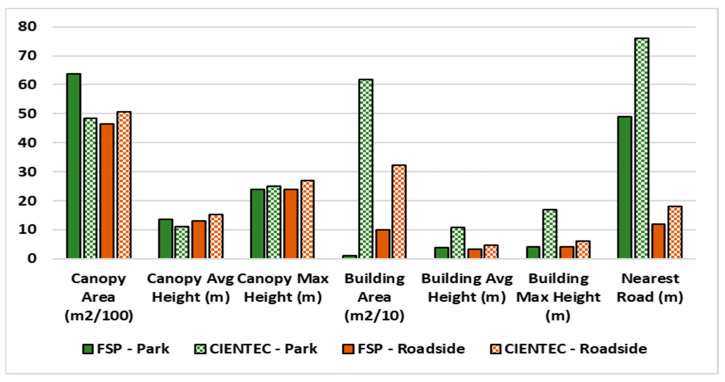
Summary of characteristics of Park (green) and Roadside (brown) sensors for FSP (solid) and CIENTEC (gridded) sites.

The site characteristics, specifically vegetation area, tree crown height, building area, building height and distance to the nearest road, were calculated for a 50 m radius around each site (see Figure 2). The vegetation and building area can be understood as proxies for the presence (or absence) of GBGI features, while their respective heights are known to influence particle dispersion. Road distance represents the presence of local mobile emission sources. The lidar and road network data are made publicly available by the Municipal Government of São Paulo through the geosampa (https://geosampa.prefeitura.sp.gov.br/PaginasPublicas/_SBC.aspx (accessed on 23 September 2024)), a web platform that consolidates a wide variety of spatialized data for the city.

It should be noted that while the closest roads to both sites are classified as arterial, the FSP site experiences higher vehicle traffic. The two nearest roads at FSP meet to form a major intersection that experiences some traffic congestion throughout the day, which itself increases local emissions [25].

### 2.2. Instrumentation

The sensors used in this study are PurpleAir Flex Air Quality Monitors, which use a pair of Plantower PMS-6003 laser particle counters (Plantower Technology, Jiangxi, China) and one BME688 Bosch Sensortec instrument (Robert Bosch GmbH, Baden-Württemberg, Germany) for meteorological readings (temperature, humidity, and pressure). Unless modified otherwise, the sensor collects data at 2 min intervals. The PurpleAir sensors have been widely used in previous studies [26,27]; however, due to variations in their performance in the field, extensive quality assurance is required. Prior to installation, the performance of the sensors deployed in this study was validated via colocation within a chamber at the Global Centre for Clean Air Research (GCARE) laboratory. The collocation experiments took place during the period from 22 to 24 May 2023. A variety of scenarios were run, simulating a range of temperature, humidity and particulate matter concentration settings. The sensors showed adequate performance for particulate matter and climate readings; therefore, no calibration of the sensor readings was performed in this study. Results from the validation experiments can be found in the Supplementary Materials. The PurpleAir sensors use laser particle counters and convert these readings into PM concentrations for PM_1_, PM_2.5_ and PM_10_. This conversion process is proprietary, but the final readings generated by the sensors are available for two correction factors: CF = 1 and CF = atm. The CF = 1 correction factor is recommended for indoor monitoring (factory conditions), whereas the CF = atm version is recommended for outdoor uses (atmospheric conditions). The CF = atm data were used for the comparison with the reference instrument.

### 2.3. Data Collection

The sensors at FSP were installed in June 2023. At CIENTEC, the sensors were installed in July (roadside) and September (park) 2023. The sensors remained in place throughout the period analyzed; however, there were some disruptions that created data gaps. While the sensors transmit data via Wi-Fi, data were obtained from the backup SD cards that log the readings continuously, thus reducing missingness. The final raw data considered in this study were collected in May 2024. The final period considered for analysis runs from 30 June 2023 to 4 April 2024.

### 2.4. Quality Assurance and Control

We applied several data cleaning steps to remove possibly non-representative readings and ensure agreement between sensor channels. Firstly, we applied a Limit of Detection (LOD) of 1.5 µg/m^3^. This is comparable with, but slightly more restrictive than, the LOD values derived by Wallace et al., 2021 [28]. We also removed readings above 95% humidity. No readings were outside the sensor’s functional temperature range (−17 to 54 °C). Next, to calculate hourly averages, we set a minimum threshold of 12 readings. To ensure agreement between the two laser particle counters in each sensor (referred to as the A and B channels), we applied the criteria used by Mathieu-Campbell et al., 2024 [29]. This process establishes two rules for eliminating readings, depending on the reading’s concentration (above or below 25 µg/m^3^):

When PM_2.5_ < 25 µg/m^3^, the total difference must be less than 5 µg/m^3^ and the percent difference must be less than 20%.

When PM_2.5_ > 25 µg/m^3^, only the percentage difference must be less than 20%.

Lastly, for each pair of sensors, we eliminated all resulting hourly averages that were not collected simultaneously by both sensors (park and roadside). Table 1 summarizes the data cleaning sequence and the number of readings removed during each step.

#### Background Analysis

We removed the background concentration from the hourly average readings for each sensor, allowing us to better understand the influence of local emission sources. As in Nogueira et al., 2021, we considered the overnight period from midnight to 3 a.m. to represent the background, as this period corresponds to limited vehicle traffic and stable atmospheric conditions [30]. From this selection, we calculated the 10th percentile (p10) and defined the resulting values as the background concentrations. Values below the background concentration were removed, and this value was subtracted from all remaining hourly readings. As a result, we obtained a final cleaned dataset with the background concentration removed.

### 2.5. Analysis

We considered the percentage difference between the roadside and park sensors at each site to represent the degree of possible mitigation (road value − park value = delta). We compared the degree of mitigation for the three PM fractions as well as for the climate variables temperature, humidity and pressure. The delta values were averaged by hour of day and separated into three periods. Due to the distinct pattern of variation at FSP and CIENTEC, we defined these periods differently for each site. Table 2 presents the periods used for each site.

Wind speed and direction data were obtained from Brazil’s National Institute of Meteorology (INMET), which provides wind direction at 22.5-degree intervals. We combined the hourly data from the closest INMET station with the hourly averaged sensor data. In the case of FSP, the SE-CGE–SP site, located approximately 1.5 km away, was used. For CIENTEC, the IPIRANGA station, located 4.5 km away, was used. The results of the mitigation analysis were thus separated by time of day and wind direction.

To understand the interaction between the weather data and the mitigation values, we ran multiple linear regression (MLR) models, using a stepwise algorithm to identify the models that best explain the variation in mitigation (ΔPM). This was carried out with both the final compiled data (hourly averages) and with a small dataset of only the hour-by-hour averages for each variable.

Lastly, to better understand the composition of the roadside vegetation barrier at each site, we isolated the 3D segment of the barrier and calculated the point cloud density using CloudCompare.

## 3. Results and Discussion

### 3.1. Comparison of Background Concentrations

The background analysis produced lower concentrations for the CIENTEC site, reflecting the lower overall averages for this site. Surprisingly, the background concentrations were also higher for the park sensors at each site. As mentioned, this could be a result of the vegetated areas having more biogenic precursors and better conditions for particle formation due to the increased humidity [17,18]. Table 3 shows a summary of the background concentrations and resulting overall averages for the sensors at CIENTEC and FSP.

For PM_2.5_, the final averages are 3.1 and 5.3 µg/m^3^ higher at the FSP Park and Roadside sensors, respectively.

Comparing the meteorological variables, the two sites show mixed results, as seen in Table 4. Overall, CIENTEC showed higher temperatures in the park and FSP showed higher road temperatures. This finding is surprising, given that CIENTEC is surrounded by such an expansive forested area. This could be explained by the fact that the CIENTEC sensor is situated higher on its hosting structure and is, therefore, largely exposed to direct sunlight at certain periods throughout the day. The higher humidity at the CIENTEC site shows the effect of the larger forest surrounding this site compared to FSP. The lower background and average PM values indicate more robust benefits for this expansive urban park compared to the small patch of vegetation at the FSP site. There is a nominal difference between the average pressure values for the sites; the difference could reflect the relative heights of each sensor.

#### Temporal Variation

**Figure 3:**
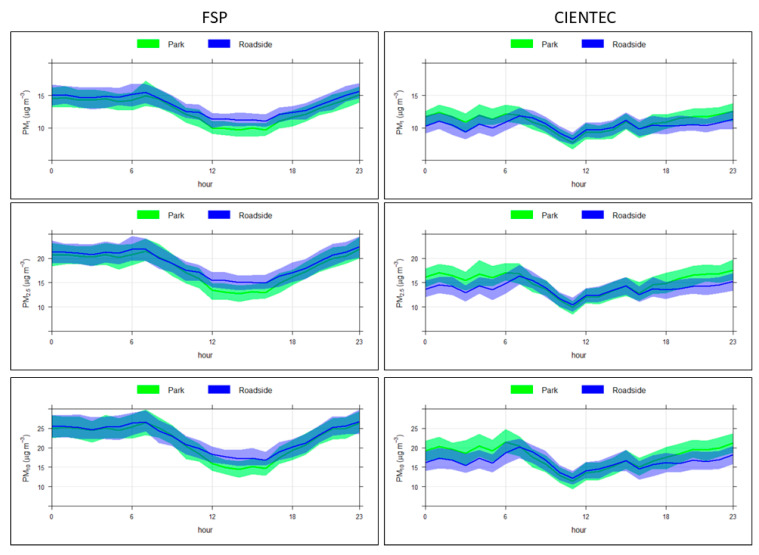
Hourly variations in concentrations of PM_1_ (**top**), PM _2.5_ (**middle**) and PM_10_ (**bottom**) for the FSP (**left**) and CIENTEC (**right**) sites.

Figure 3 shows the hourly variation in PM concentrations. Both sites display a typical diurnal trend that is likely influenced by local traffic frequency [31].

Concentrations are generally higher during the overnight period, likely owing to less favorable dispersion conditions during this period, namely the lower mixing height [32,33]. After the morning peak, concentrations steadily decrease until reaching their average daily minimum—this transition occurs earlier at the FSP site, which led us to different period of day definitions for the two sites.

### 3.2. Delta Calculation/Mitigation Analysis

Table 5 shows the results of the mitigation (delta) analysis. PM concentrations are consistently lower at the FSP park sensor (positive ΔPM), whereas the positive delta value for PM is only seen during the daytime period for CIENTEC. The delta values for PM also appear to increase with size fraction, which may reflect (i) higher fallout of heavier particles over the distance from the road and (ii) more effective barrier and deposition protection from vegetation for these size fractions.

The temporal variation in ΔPM is provided in Table S1. The delta value varies throughout the day at each site. At both sites, the difference tends towards zero or negative range at night, whereas during the daytime, the value is generally positive (meaning lower concentrations at the park sensor). The transitions from negative to positive values happen at different times, occurring slightly earlier at the CIENTEC site (8 a.m. and 3 p.m.) compared to the FSP site (10 a.m. and 6 p.m.). It is important to consider that the daytime period corresponds to a higher effect of local emission sources and is the period we would expect exposure to occur. In this sense, we can say that at both sites, the vegetation appears to provide daytime protection to potentially exposed populations.

The delta results for the weather data indicate that the FSP site protects against high temperatures and low humidity, while this is not seen at CIENTEC during the day. The pressure levels vary nominally at both sites.

The lower PM concentrations at the CIENTEC site compared to the FSP site indicate that the much larger forested area acts as a mitigating buffer against PM. This effect is likely magnified by the absence of local emissions sources compared to the FSP site. The lower temperature and higher humidity for CIENTEC also highlight how the large forested area acts as a buffer against climate stressors.

#### Vegetation Barrier Porosity

**Figure 4:**
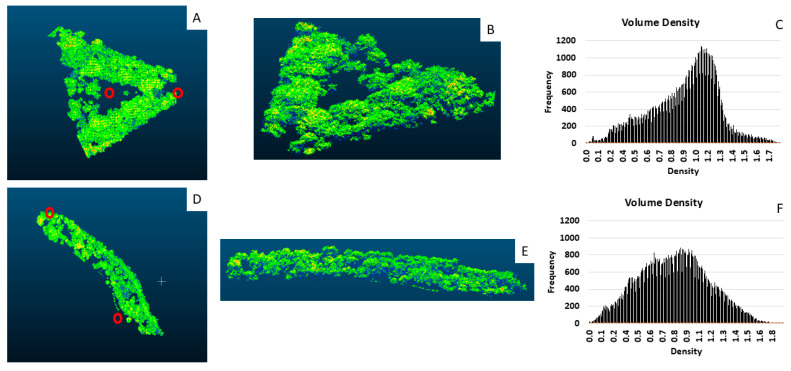
Top (**A**,**D**) and side (**B**,**E**) views of vegetation barriers at each site and point cloud density distribution (**C**,**F**) for FSP (**bottom**) and CIENTEC (**top**). The red circles indicate the sensor locations for the top view.

As mentioned previously, the CIENTEC park sensor is situated in a more exposed area compared to the FSP park sensor, which could help explain the less effective PM and climate protection. Another possible explanation for this could be the structure of the roadside vegetation barrier for this site, which appears to be more porous. Figure 4 shows 3D representations of the vegetation barriers that are road-adjacent, as well as the point density distribution for these segments.

Whereas the nearby road at CIENTEC is roughly NW to SE, the FSP site has two adjacent roads on its north and south sides, so we considered all vegetation within this triangle as part of the vegetation barrier. The 3D rendering of the lidar data appears to show a more porous barrier at the CIENTEC site, with visible gaps from the top–down and side views. We can see that the peak of the density curve occurs at a higher value for the FSP site, indicating that this barrier is less porous compared to the CIENTEC barrier. This could lead to a less effective barrier and may explain the discrepancy in ΔPM values between the sites.

### 3.3. Effect of Wind Speed and Direction

**Figure 5:**
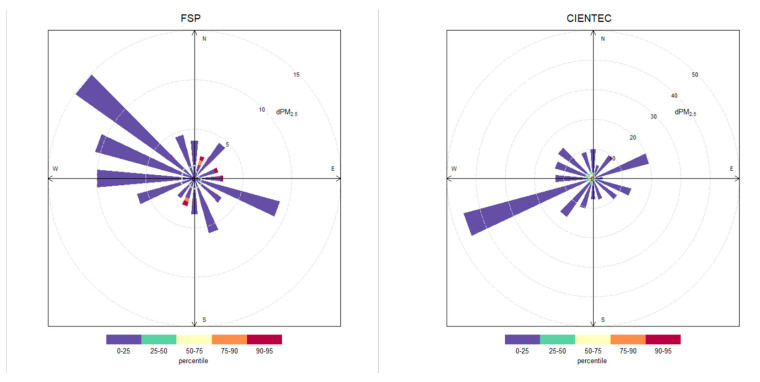
Percentile rose distribution of the difference between road and park PM_2.5_ concentrations at FSP (**left**) and CIENTEC (**right**) sites.

Figure 5 shows the distribution of the ΔPM_2.5_ by percentile and wind direction, revealing the wind direction at which the ΔPM_2.5_ values are maximized for each site.

Overall, westerly winds appear to improve the PM_2.5_ mitigation at both sites, specifically northwesterly winds for FSP and southwesterly for CIENTEC. This represents a roughly crosswind scenario (refer to Figure 1) at both sites and, thus, could reflect the impact of vehicle emissions on the roadside sensor concentrations. Averaging the percentage of ΔPM_2.5_ by wind direction produces similar results, with the highest values occurring between 225 and 270 (20–32% reduction for FSP and 5–9% reduction for CIENTEC) (see Table S2).

### 3.4. Combined Effect of Variables

The stepwise regression modeling aims to determine which variables have the strongest influence on ΔPM_2.5_. This process produced low R2 values when considering the full datasets for each site. However, when we reduced the data to hour-by-hour averages for each variable, the R2 increased significantly. Table 6 shows the variables and their respective coefficients for each site.

The ΔPM_2.5_ is influenced by different factors at each site. Whereas windspeed promotes mitigation at the FSP site, it appears to have the opposite effect at the CIENTEC site; this also applies to temperature and pressure.

Overall, the results show that as a large urban forest, the CIENTEC site provides robust ecological benefits such as increased humidity and lower background and average PM concentrations. Additionally, the vegetation at both sites appears to reduce daytime PM concentrations (although at different times of day); overall, these benefits are more limited at the CIENTEC site, which may be due to a differing roadside vegetation barrier structure. These benefits are augmented by favorable wind conditions. Other climate factors appear to affect the mitigation differently at each site. This, in turn, has positive impacts on public health, as epidemiological and toxicological studies have shown associations between both chronic and long-term exposure to PM and a plethora of adverse health effects, including airway damage and cardiopulmonary disorders [34]. Unfavorable thermal comfort conditions have also been associated with mortality for circulatory and respiratory diseases, as extreme heat can lead to vasodilation to dissipate heat and maintain thermal equilibrium, potentially leading to acute cardiovascular events [35].

## 4. Conclusions

As we enter a new era of uncertainty concerning the local impacts of climate change, it is increasingly important for urban adaptation policies to be rooted in experimental evidence. Nature-based solutions such as GBGIs offer the possibility of various co-benefits such as mitigation, adaptation and reduced exposure to air pollution. GBGIs are especially important tools in the context of climate and air pollution mitigation policies, aiming to bring climate justice to vulnerable populations. However, the interaction between these can produce mixed results and needs to be better understood. The results of this study reinforce the important role that green infrastructure can play in mitigating environmental and climate impacts. It also shows how different site characteristics can affect the magnitude of this mitigating effect. This information should be taken into account when designing green–blue infrastructure so as to maximize benefits and reduce population exposure. This study reinforces that low-cost sensors show promising results and large potential use for cities in low-income settings where official monitoring stations are scarce or non-existent.

## Figures and Tables

**Table 1:** Overview of data cleaning criteria and number of readings removed at each step.

Criteria	Number of Readings Removed (Percentage of Total)
*Initial total: 765788 2-min readings*	
Detection limit PM_2.5_ > 1.5 µg/m^3^	75,695 (10%)
Relative humidity > 95%	120 (0.02%)
*Total hourly readings: 24718*	
At least 12 2-min readings to form hourly averages	4178 (17%)
A-B channel criteria	1029 (4%)
Simultaneous readings	3635 (15%)

This process resulted in 7853 hourly average readings (3489 for CIENTEC and 4364 for FSP).

**Table 2:** Separation of period of day for each site.

Period of Day	FSP	CIENTEC
Overnight	00 h to 09 h	00 h to 07 h
Daytime	10 h to 18 h	08 h to 15 h
Evening	19 h to 23 h	16 h to 23 h

**Table 3:** Background and average concentrations (in µg/m^3^) for sensors at the CIENTEC and FSP sites.

	CIENTEC				FSP			
	Park		Roadside		Park		Roadside	
	Back	Avg	Back	Avg	Back	Avg	Back	Avg
PM_1_	3.8	10.9	3.6	10.3	5.2	12.8	4.7	13.6
PM_2.5_	5.1	15.0	5.0	13.8	6.8	18.1	6.0	19.0
PM_10_	6.5	17.7	6.5	16.3	8.0	21.8	7.1	22.7

**Table 4:** Average, median and standard deviation of meteorological parameters for each site.

	CIENTEC						FSP					
	Park			Roadside			Park			Roadside		
	Avg	Med	SD	Avg	Med	SD	Avg	Med	SD	Avg	Med	SD
**Temperature (°C)**	28.6	27.7	4.8	29.1	28.2	4.1	27.6	26.5	5.6	30.0	29.2	5.8
**Humidity (%)**	54.1	57.5	11.6	52.8	55.7	9.2	50.5	54.3	12.8	44.1	46.3	11.6
**Pressure (mbar)**	924.1	924.0	2.8	926.2	926.1	2.8	923.6	923.3	3.8	923.7	923.3	3.8
**Wind Direction (°)**	145.8	112.0	102.3	145.8	112.0	102.3	144.9	112.0	108.5	144.9	112.0	108.5
**Wind Speed (m/s)**	0.2	0.0	0.6	0.2	0.0	0.6	1.1	0.7	1.2	1.1	0.7	1.2

**Table 5:** Average difference between road and park sensors at FSP and CIENTEC per period of day.

	FSP			CIENTEC		
	PM_1_	PM_2.5_	PM_10_	PM_1_	PM_2.5_	PM_10_
**Overnight**	0.47 (10%)	0.50 (11%)	0.50 (11%)	−1.2 (−15%)	−2.2 (−20%)	−2.7 (−21%)
**Daytime**	1.12 (22%)	1.7 (26%)	1.7 (27%)	0.42 (11%)	0.33 (10%)	0.66 (12%)
**Evening**	0.59 (13%)	0.65 (13%)	0.65 (13%)	−0.86 (−10%)	−1.8 (−14%)	−2.1 (−15%)
	**Temp**	**Hum**	**Press**	**Temp**	**Hum**	**Press**
**Overnight**	2.9 (10%)	−7.5 (−16%)	0.04 (0%)	0.72 (3%)	−2.3 (−4%)	2.1 (0%)
**Daytime**	1.8 (6%)	−4.7 (−11%)	0.06 (0%)	−0.3 (0%)	0.9 (3%)	2.2 (0%)
**Evening**	2.6 (10%)	−7.1 (−14%)	0.03 (0%)	0.96 (4%)	−2.4 (−4%)	2.2 (0%)

**Table 6:** Variables and their respective coefficients of stepwise-derived equations (T = temperature; H = humidity; P = pressure; WD = wind direction; WS = wind speed).

		Variables and Coefficients																R2
		Park Sensor						Roadside Sensor						Wind Data				
**FSP**	**Full Data**	T	2.75	H	–0.32	P	0.26	T	–2.80	H	NA	P	NA	WD	0.01	WS	4.05	0.08
	**Hour-by-hour**	T	14.30	H	3.11	P	172.90	T	–5.67	H	NA	P	–171.40	WD	0.22	WS	NA	0.93
**CIENTEC**	**Full Data**	T	–2.37	H	–2.85	P	–9.30	T	4.21	H	2.65	P	9.49	WD	0.02	WS	–2.32	0.26
	**Hour-by-hour**	T	NA	H	–1.35	P	NA	T	NA	H	NA	P	2.41	WD	0.24	WS	–26.88	0.94

## Data Availability

The PurpleAir sensor data can be downloaded from the PurpleAir platform: https://www2.purpleair.com/ (accessed on 4 February 2025).

## Supplementary Materials

The following supporting information can be downloaded at: https://www.mdpi.com/article/10.3390/ijerph22020306/s1, Table S1: Variation in ΔPM by site, pollutant and hour of day; Table S2: Validation Experiment Results.
