# Supplementary material for: Exploring Climate and Air Pollution Mitigating Benefits of Urban Parks in Sao Paulo Through a Pollution Sensor Network
Source: Int J Environ Res Public Health. 2025 Feb 18;22(2):306. doi: 10.3390/ijerph22020306 (PMC11854963; doi:10.3390/ijerph22020306)
Supplement: Supplementary file 1 [file ijerph-22-00306-s001.zip › ijerph-3410725-supplementary.pdf]

**Supplementary Material**

Table S1 Variation in ΔPM by site, pollutant and hour of day.

| Hour | FSP PM1 | CIENTEC PM1 | FSP PM25 | CIENTEC PM25 | FSP PM10 | CIENTEC PM10 |
|---|---|---|---|---|---|---|
| 0 | 0.10 | -1.47 | -0.11 | -2.35 | -0.33 | -2.74 |
| 1 | -0.01 | -1.45 | -0.24 | -2.37 | -0.55 | -2.75 |
| 2 | 0.00 | -1.33 | -0.24 | -2.11 | -0.52 | -2.45 |
| 3 | -0.08 | -1.58 | -0.32 | -2.45 | -0.66 | -2.82 |
| 4 | -0.08 | -1.45 | -0.34 | -2.37 | -0.62 | -2.80 |
| 5 | 0.23 | -1.49 | 0.09 | -2.41 | -0.04 | -2.83 |
| 6 | 0.46 | -1.34 | 0.35 | -2.13 | 0.24 | -2.45 |
| 7 | 0.14 | -0.26 | -0.23 | -0.52 | -0.54 | -0.22 |
| 8 | -0.25 | 0.41 | -0.81 | 0.58 | -1.26 | 1.34 |
| 9 | -0.12 | 0.23 | -0.53 | 0.31 | -0.87 | 0.95 |
| 10 | 0.10 | 0.18 | -0.14 | 0.26 | -0.27 | 0.73 |
| 11 | 0.47 | 0.23 | 0.52 | 0.36 | 0.56 | 0.78 |
| 12 | 0.86 | 0.13 | 1.26 | 0.19 | 1.51 | 0.57 |
| 13 | 1.07 | 0.17 | 1.57 | 0.20 | 1.88 | 0.59 |
| 14 | 1.04 | 0.15 | 1.49 | 0.11 | 1.76 | 0.44 |
| 15 | 0.82 | 0.04 | 1.11 | -0.13 | 1.31 | 0.10 |
| 16 | 0.89 | -0.18 | 1.19 | -0.44 | 1.35 | -0.32 |
| 17 | 0.56 | -0.40 | 0.67 | -0.80 | 0.69 | -0.73 |
| 18 | 0.34 | -0.73 | 0.27 | -1.25 | 0.16 | -1.28 |
| 19 | 0.16 | -1.22 | -0.05 | -2.00 | -0.21 | -2.25 |
| 20 | 0.12 | -1.35 | -0.16 | -2.24 | -0.43 | -2.49 |
| 21 | 0.16 | -1.47 | -0.06 | -2.36 | -0.28 | -2.68 |
| 22 | 0.23 | -1.45 | 0.01 | -2.37 | -0.21 | -2.74 |
| 23 | 0.10 | -1.37 | -0.15 | -2.30 | -0.39 | -2.72 |

Table S2. shows a variety of scenarios in which the PA sensors were compared to a reference instrument by pollutant, including the temperature and humidity ranges for each experiment.

Table S2. Validation Experiment Results

| Temperature Range | Humidity Range | Parameter | R Value Range |
|---|---|---|---|
| 11-14 | 60-76 | PM1 | 0.90-0.95 |
| 9-11 | 25-40 | PM1 | 0.79-0.96 |
| 9-11 | 40-60 | PM1 | 0.98-1.00 |
| 20-22 | 29-40 | PM1 | 0.88-0.98 |
| 20-22 | 40-60 | PM1 | 0.92-0.99 |
| 20-22 | 60-80 | PM1 | 0.87-0.98 |
| 26-28 | 50-75 | PM1 | 0.97-0.98 |
| 26-28 | 30-50 | PM1 | 0.99-1.00 |
| 11-14 | 60-76 | PM2.5 | 0.87-0.92 |
| 9-11 | 25-40 | PM2.5 | 0.79-0.96 |
| 9-11 | 40-60 | PM2.5 | 0.99-1.00 |
| 20-22 | 29-40 | PM2.5 | 0.88-0.98 |
| 20-22 | 40-60 | PM2.5 | 0.92-0.99 |
| 20-22 | 60-80 | PM2.5 | 0.85-0.97 |
| 26-28 | 50-75 | PM2.5 | 0.97-0.98 |
| 26-28 | 30-50 | PM2.5 | 0.99-1.00 |
| 11-14 | 60-76 | PM10 | 0.85-0.90 |
| 9-11 | 25-40 | PM10 | 0.79-0.96 |
| 9-11 | 40-60 | PM10 | 0.99-1.00 |
| 20-22 | 29-40 | PM10 | 0.87-0.98 |
| 20-22 | 40-60 | PM10 | 0.92-0.99 |
| 20-22 | 60-80 | PM10 | 0.86-0.97 |
| 26-28 | 50-75 | PM10 | 0.94-0.97 |
| 26-28 | 30-50 | PM10 | 0.99-1.00 |
